# Bridging the macro to micro resolution gap with angiographic optical coherence tomography and dynamic contrast enhanced MRI

**DOI:** 10.1038/s41598-022-07000-1

**Published:** 2022-02-24

**Authors:** W. Jeffrey Zabel, Nader Allam, Warren D. Foltz, Costel Flueraru, Edward Taylor, I. Alex Vitkin

**Affiliations:** 1 Department of Medical Biophysics, University of Toronto, Toronto, Canada; 2 Radiation Medicine Program, Princess Margaret Cancer Centre, Toronto, Canada; 3 Department of Radiation Oncology, University of Toronto, Toronto, ON Canada; 4 National Research Council Canada, Information Communication Technology, Ottawa, Canada

**Keywords:** Biomarkers, Cancer imaging, Cancer microenvironment, Cancer therapy, Tumour angiogenesis, Tumour biomarkers, Oncology, Tumour heterogeneity, Applied optics, Magnetic resonance imaging, Three-dimensional imaging, Experimental models of disease, Preclinical research, Translational research, Imaging and sensing, Microscopy, Wide-field fluorescence microscopy, Optics and photonics, Biophotonics

## Abstract

Dynamic contrast enhanced magnetic resonance imaging (DCE-MRI) is emerging as a valuable tool for non-invasive volumetric monitoring of the tumor vascular status and its therapeutic response. However, clinical utility of DCE-MRI is challenged by uncertainty in its ability to quantify the tumor microvasculature ($\mu \mathrm{m}$ scale) given its relatively poor spatial resolution (mm scale at best). To address this challenge, we directly compared DCE-MRI parameter maps with co-registered micron-scale-resolution speckle variance optical coherence tomography (svOCT) microvascular images in a window chamber tumor mouse model. Both semi and fully quantitative (Toft’s model) DCE-MRI metrics were tested for correlation with microvascular svOCT biomarkers. svOCT’s derived vascular volume fraction (VVF) and the mean distance to nearest vessel ($\overline{\mathrm{DNV} }$) metrics were correlated with DCE-MRI vascular biomarkers such as time to peak contrast enhancement ($r=-0.81$ and $0.83$ respectively, $P<0.0001$ for both), the area under the gadolinium-time concentration curve ($r=0.50$ and $-0.48$ respectively, $P<0.0001$ for both) and ${k}_{trans}$ ($r=0.64$ and $-0.61$ respectively, $P<0.0001$ for both). Several other correlated micro–macro vascular metric pairs were also noted. The microvascular insights afforded by svOCT may help improve the clinical utility of DCE-MRI for tissue functional status assessment and therapeutic response monitoring applications.

## Introduction

Tumor cells require oxygen and nutrients to survive, which are supplied by the tumor vascular network. This network is abnormal and malformed with leaky and tortuous vessels in comparison to normal tissue. The malformed nature of the tumor vasculature often results in poorly oxygenated or hypoxic regions which are more likely to metastasize and are also more resistant to common cancer treatments such as radiation therapy (RT) and chemotherapy. Such characteristics of the tumor vasculature are typically highly heterogenous spatially (on the scale of ~ 100 $\mu \mathrm{m}$ or less)^1^ and temporally^2^.

Given the strong dependence of tumor cell survival and treatment resistance on the vascular network, there has been a significant effort to develop new cancer treatments that target the tumor vasculature. For example, in the radiation oncology field, clinicians have explored the administration of a vascular endothelial growth factor (VEGF) blockade with the goal of ‘normalizing’ the vasculature to improve oxygen and nutrient delivery before RT^3^, or administration of vascular disrupting agents that block angiogenesis after RT to starve the tumor of nutrients and oxygen^4^. Multiple studies also suggest that hypofractionated RT (at doses > 8–10 Gy/fraction) depends on microvascular ablation in addition to tumor cellular DNA damage as part of its mechanism of action^5–7^.

Despite the promising early results of these therapies, optimal scheduling and dosages for concurrent administration with conventional treatment modalities (such as chemotherapy and radiation therapy) has yet to be realized^3,8,9^. Non-invasive imaging of the detailed structure and function of the tumor vasculature may facilitate therapy personalization during the treatment planning stage, rapid adaptation throughout the course of treatment, and prognosis post-treatment^3,8,9^.

Optical microangiography plays an important role in preclinical^6,10–14^ and selected clinical^15,16^ studies of the vasculature. Speckle variance optical coherence tomography (svOCT) is a functional extension of conventional structural OCT that allows for high resolution imaging of tumor microvasculature^10^. svOCT has advantages over many other intravital microscopy techniques in that it does not require exogenous contrast agents, can image the vasculature in 3D to a depth of 1–3 mm, and is relatively cheap and fast. Most importantly, svOCT’s vascular contrast is excellent and its resolution affords capillary imaging. svOCT has been used in studies to quantify differences between normal and tumor vasculature^11^ as well as to study the response of the vasculature to radiation^6,15^ and photodynamic therapies^16^. Despite its excellent ability to visualize and quantify the volumetric maps of tissue blood vessels down to the capillary level and selected therapy-monitoring applications in patients^15,16^, clinical implementation of this technology in a wide variety of anatomical sites is hindered by its shallow penetration depth^17^. An alternate imaging modality with deep-tissue vascular imaging capabilities must be considered for wider clinical implementation.

Magnetic resonance imaging (MRI) is already prevalent in cancer care, given its excellent soft tissue contrast and ability for large field-of-view imaging in 3D with excellent penetration depth and avoidance of harmful ionizing radiation. Cancer therapies can in principle be monitored longitudinally via MRI biomarkers that report on tumor treatment response, including using perfusion-sensitive techniques such as dynamic contrast enhanced (DCE) approaches^18–20^. DCE-MRI involves the bolus administration of a gadolinium (Gd) contrast agent followed by a time series imaging of its tissue accumulation and clearance. Analysis of Gd contrast enhancement kinetics can offer insight into the function, and perhaps underlying structure of the vasculature^13,14,21–24^, and its response to vascular targeting therapies^20^.

Despite the promise of DCE-MRI for predicting and monitoring cancer treatment response, questions have been raised on its ability to accurately assess and quantify the tumor microvasculature ($\mu \mathrm{m}$ scale) given the relatively coarse spatial resolution of clinical MRI scanners (mm scale at best)^17^. To address this concern, preliminary studies have compared DCE-MRI parameters with histology^21–24^ and intravital microscopy^13,14^, and have found some correlations between these modalities. However, both histological comparisons^21–24^ and intravital microscopy studies^13,14^ suffer from poor co-registration with MR images and are limited by their simple 2D analysis of the vascular network. Here we attempt to fully analyze and address the ‘macro-to-micro resolution gap’ by directly correlating high resolution 3D *in-vivo* volumetric ‘ground truth’ svOCT images of the tumor vasculature with semi-quantitative and quantitative vascular metrics derived from lower resolution MR-contrast enhancement time series imaging in the same animals. Through these correlations, we hope to determine whether microvascular information can be gleaned from coarser-resolution DCE-MRI datasets, thereby increasing its available information content and potentially enhancing its clinical value.

## Materials and methods

### Animal model and window chamber design

**Figure 1:**
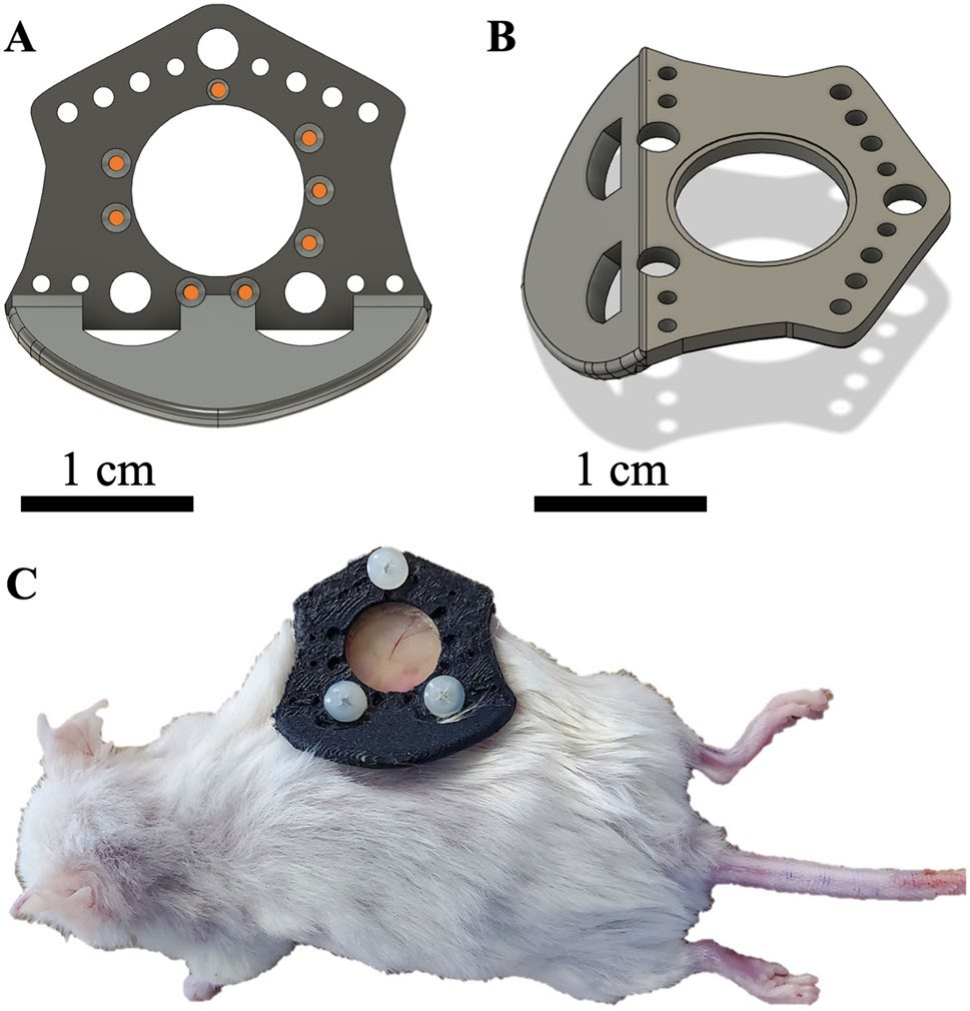
MR-compatible plastic window chamber mouse model. 3D rendering of top (**A**) and bottom (**B**) surface of MR compatible plastic window chamber designed in Autodesk Fusion 360 CAD software version 2.0.12160 (Autodesk Inc., San Rafael, CA, USA). Divots for fiducial marker placement are marked by the orange circles in (**A)**. (**C)** Plastic window chamber on a tumor bearing mouse.

All animal procedures were performed in accordance with appropriate standards under a protocol approved by the University Health Network Institutional Animal Care and Use Committee in Toronto, Canada (AUP #3256). The reporting in this manuscript follows the ARRIVE guidelines. The radioresistant and immunocompromised NOD-Rag1^null^IL2r $\gamma$ ^null^ (NRG) mouse strain was chosen for this study. Seven mice were subcutaneously inoculated with ~${10}^{5}$ human pancreatic cancer cells (BxPC-3 cell line) transfected with fluorescent DsRed to report cellular viability. Two mice with no tumor (bare skin only) were used as ‘normal’ vasculature controls. MR compatible window chambers were designed using Autodesk Fusion 360 CAD software version 2.0.12160 (Autodesk Inc., San Rafael, CA, USA) and 3D printed using a carbon fiber and nylon blend thermoplastic (Filaments Inc, Toronto, ON, CA). The window chamber was surgically sutured to the dorsal skin folds (Fig. 1) when the tumor reached ~ 3–5 mm in diameter (~ 3–4 weeks post inoculation). Window chamber installation was intentionally performed *after* tumor inoculation so that the initial tumor growth dynamics and its microvascular development were not altered^25^. The window chambers had eight ‘divots’ on the top surface for placement of fiducial markers (Tear-Gel, Bausch & Lomb, Laval, QC, CA) to facilitate MRI to svOCT image co-registration (Fig. 1A, orange circles). For both optical and MR imaging, mice were anesthetized using 5% isoflurane and maintained with 2% isoflurane via a nose cone mask.

### Optical imaging: experimental setup

OCT images were acquired using a previously described swept source OCT system based on a quadrature interferometer^26^ allowing for acquisition of the full complex interferometric signal to suppress the complex conjugate artifact. The sample arm of the quadrature interferometer contained a semiconductor optical amplifier (SOA) with a gain of 35 dB to boost the back reflected signal from the tissue. The SOA had the same center wavelength and bandwidth as the laser source (1,300 nm and 105 nm FWHM, respectively). Polarization controllers were used to minimize the differences between the shape of normalized light spectra in the reference arm and after the SOA. The A-scan rate of the OCT imager was 20 kHz. Two detector outputs were digitized using a data acquisition card with 16-bit resolution and sampling rate of 250 MS/s. The resultant axial and lateral resolutions in air were 8 $\mu \mathrm{m}$ and 15 $\mu \mathrm{m}$, respectively.

**Figure 2:**
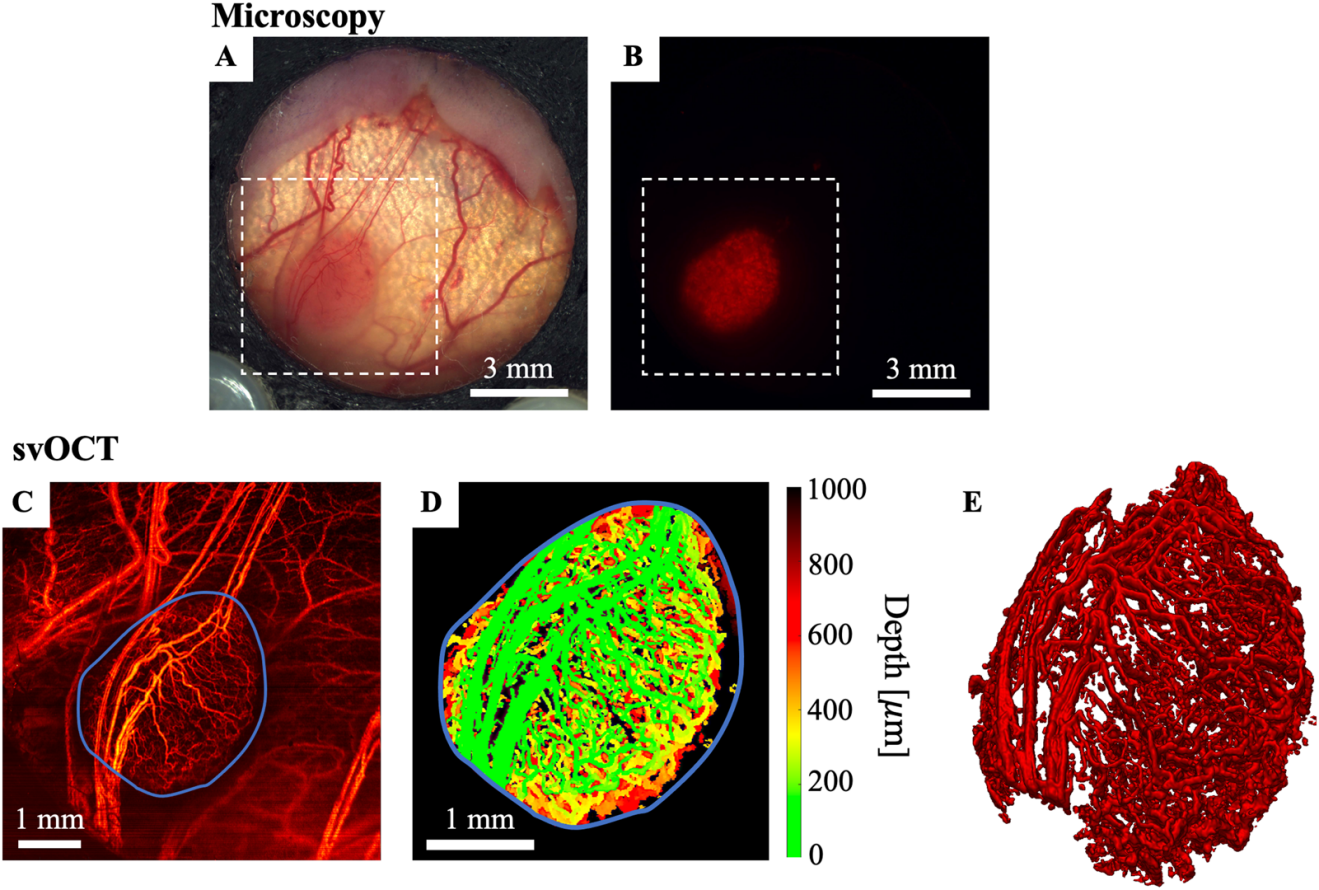
Brightfield, fluorescence, and svOCT imaging of a well vascularized tumor. (**A)** Brightfield image of window chamber with white dotted line indicating the field of view of the svOCT image. (**B)** Corresponding DsRed fluorescence image to indicate tumor cell viability. (**C)** svOCT average intensity projection with tumor boundary delineated by the blue line. (**D)** Segmented depth encoded vasculature within blue tumor boundary line. (**E**) 3D rendering of segmented tumor vasculature. (**C**)–(**E**) were generated using MATLAB R2020A software (MathWorks, Inc., Natick, MA, USA).

OCT volumetric images were acquired with a $6\times 6$ mm^2^ field of view (FOV), by stitching together laterally adjacent $3\times 6$ mm^2^ scans. The subdivided acquisition of the FOV reduces bulk tissue motion artifacts by decreasing the amplitude of motion of the OCT’s scanning-mirror galvanometer, thus reducing the inter-frame time (the time between scan repetitions at a given location). Each B-scan consisted of 400 A-scans and was performed 8 times per location (25 ms apart) to enable speckle variance processing^10^. This repeat sequence and temporal spacing were previously determined to result in sufficiently high speckle variance SNR and fast decorrelation in blood microvasculature compared to other physiological motions of surrounding tissue^27^. Hence enhanced contrast for image voxels containing blood is obtained via interframe speckle variance calculations. Tumor cell brightfield (Fig. 2A) and DsRed fluorescence (Fig. 2B) imaging were also performed for localization and viability assessment, respectively, using an epifluorescence microscope (Leica Microsystems MZ FLIII, Richmond Hill, ON, CA).

### Optical imaging: speckle variance processing and microvascular metric extraction

OCT data were analyzed using in-house built software written in MATLAB R2020A (MathWorks, Inc., Natick, MA, USA). Speckle variance processing was used to obtain 3D images of the tumor microvasculature^10^. The interframe intensity variance ${SV}_{xyz}$ across $N$ consecutive structural OCT intensity scans ${I}_{xyzn}$ (where $xyz$ are the voxel coordinates) was calculated to obtain 3D images with enhanced vascular contrast (*N* = 8 for these experiments):

1 $${SV}_{xyz}=\frac{1}{N}\sum_{n=1}^{N}{\left({I}_{xyzn}-\frac{1}{N}\sum_{n=1}^{N}{I}_{xyzn}\right)}^{2}$$

A resultant representative 2D average intensity projection image of the microvasculature is shown in Fig. 2C. To facilitate vascular quantification, vessels were segmented from the svOCT datasets using the method proposed by Conroy *et al*^11^ (Fig. 2D and E).

Vascular quantification analysis was performed within manually drawn tumor contours. These were created by first co-registering the DsRed fluorescence and brightfield images with the svOCT vascular volumes using affine transforms guided by vascular landmarks, and thresholding the fluorescence images to create a 2D tumor contour. The 2D fluorescence tumor contour was further defined by performing manual, 3D tissue surface masking using the structural OCT scans. The 3D tissue surface mask was combined with a cylindrical projection of the 2D fluorescence tumor contour to a depth of 1 mm from the most superficial tissue layer. The combination of the 2D fluorescence contour and 3D tissue surface mask yielded the final 3D tumor contour. For the healthy mouse, vascular analysis was restricted to $3\times 3 \space{\mathrm{mm}}^{2}$ region of interest in the middle of the window chamber, similar to the 3D spatial extent of most examined tumors.

Two quantitative vascular microarchitecture metrics were then extracted from the segmented svOCT datasets. The vascular volume fraction (VVF) was defined as the proportion of vessels within the analyzed volume. A 3D Euclidean distance transform was then applied to the entire segmented vascular volume to obtain a histogram of the distances to the nearest vessel (DNV). The mean distance to the nearest vessel ($\overline{\mathrm{DNV} }$) was then defined as the average value of the DNV histogram within the analyzed volume.

### MRI: experimental setup

Same animals were imaged on a 7T preclinical MRI system (Biospec 70/30 USR, Bruker Corporation, Ettlingen, BW, DE), equipped with the B-GA12 gradient coil insert and 7.2 cm inner diameter quadrature cylindrical RF coil. Each mouse was anesthetized and positioned on a slider bed with inlaid tubes circulating water warmed to 38 $^\circ{\rm C}$ for temperature regulation. A respiratory pillow (SA Instruments, Stony Brook, NY, USA) was positioned underneath the diaphragm for respiratory monitoring, maintained at $30\pm 5$ breaths per minute. A 3D printed thermoplastic polyurethane window chamber immobilization device reduced respiratory movement and aligned the window chamber plane along the axis of the MRI bore.

**Figure 3:**
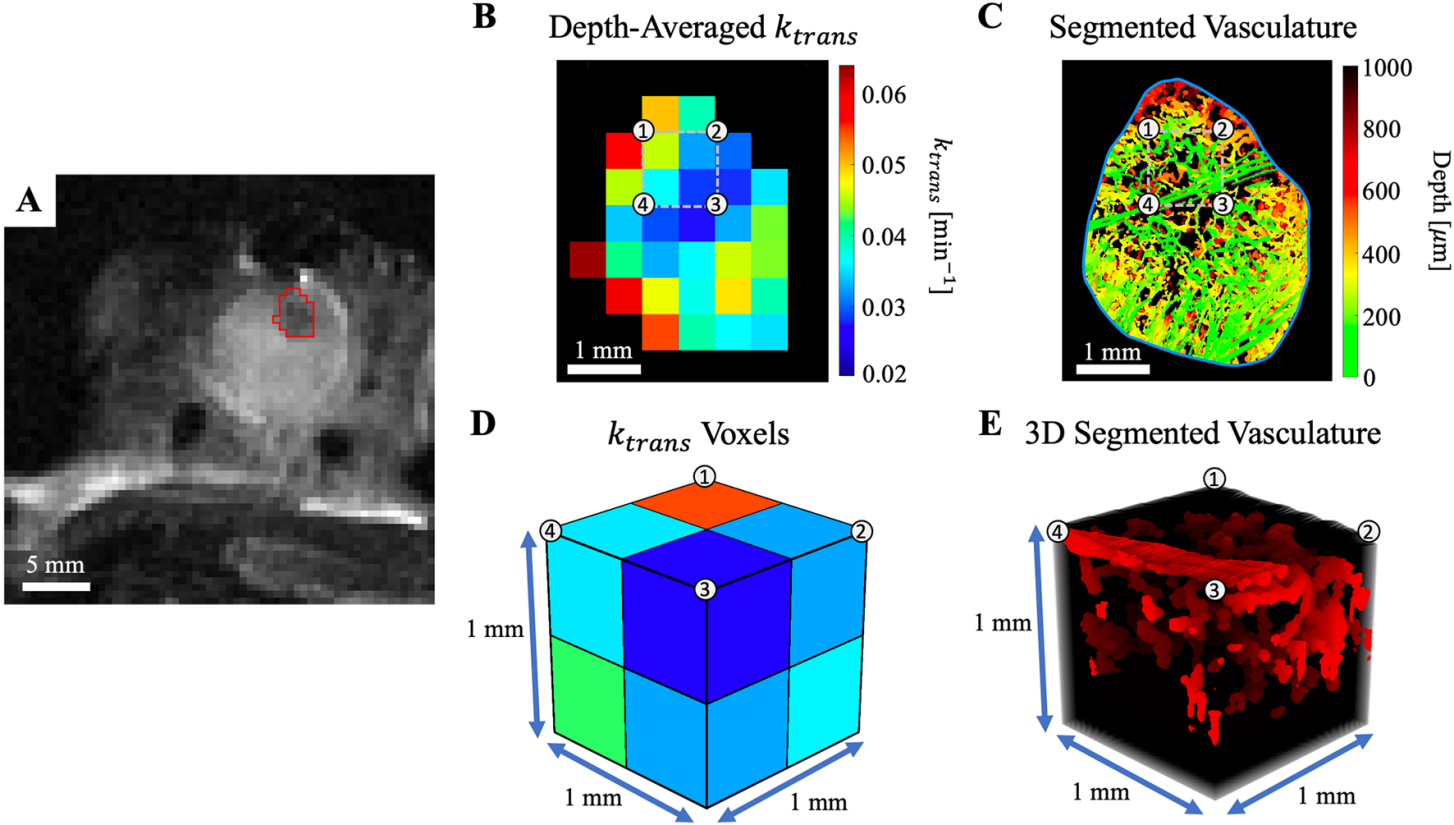
Co-registered macro DCE-MRI to micro svOCT vascular correlations. (**A)** ${T}_{2}$-weighted structural MRI scan of the window chamber with tumor delineated by the red contour. (**B)** ${k}_{trans}$ parameter map of the tumor, averaged over two depth slices (total depth of 1 mm to correspond with svOCT’s imaging penetration), in units of min^−1^ indicated by the colour bar. (**C)** svOCT segmented depth-encoded vasculature coregistered to (**B)**. The grey dotted line in (**B)** and (**C)** shows one position of the $1 \space{\mathrm{mm}}^{3}$ sliding window VOI with numbered edges that correspond to the number locations in (**D)** and (**E)**. The various semi-quantitative and quantitative MR vascular metrics in the resulting DCE-MRI voxels (8 ${k}_{trans}$ voxels in this example) (**D**) are directly compared to microvascular biomarkers derived from the corresponding svOCT 3D microvascular map (**E**). The VOI then slides throughout the delineated tumor contour, with such analysis repeated at all positions. (**B)**–(**E**) were generated using MATLAB R2020A software (MathWorks, Inc., Natick, MA, USA).

Imaging consisted of orthogonal 2D ${T}_{2}$-weighted acquisitions to guide localization of the window chamber in the slice plane, followed by quantitative ${T}_{1}$ mapping and DCE imaging. All acquisitions shared matching geometric features ($32\times 32$ mm field-of-view with $64\times 64$ matrix for $0.5\times 0.5$ mm in-plane resolution; 5 contiguous imaging slices, with only the top two subsequently analyzed for correlation with svOCT; 0.5 mm slice thickness). ${T}_{2}$-weighted images (Fig. 3A) were acquired using 2D-RARE (echo time TE = 25 ms; repetition time TR = 2500 ms; 25 s per orientation). ${T}_{1}$ maps were generated using 2D-RARE images acquired at variable repetition time (TE = 7 ms; TR = 350, 500, 750, 1000, 1500, 2500, and 4000 ms; 8 min 28 s). Dynamic contrast enhancement was monitored using a time-series of 2D spin-echo RARE images (TE = 8.1 ms; TR = 200 ms; flip angle = 90°; temporal resolution = 12.8 s; 188 repetitions; total monitoring time = 40 min 6 s). 0.75 mmol/kg body weight Gadovist (Gd-D03A-Butrol, Bayer AG, Leverkusen, NRW, DE) was injected over 10 s via tail vein after completion of five image repetitions using an automated MR-compatible syringe pump (PHD 2000, Harvard Apparatus, Holliston, MA, USA).

### Dynamic contrast enhanced MRI: data analysis

For DCE-MRI analysis, the raw MRI signal enhancement was converted to gadolinium concentrations for each imaging timepoint (${C}_{t}$) using a standard equation for MRI contrast enhancement:

2 $${C}_{t}=\frac{\frac{1}{{T}_{1}}-\frac{1}{{T}_{\mathrm{1,0}}}}{r}$$

where ${T}_{\mathrm{1,0}}$ is the longitudinal relaxation time assessed via ${T}_{1}$ mapping, and $r=4.2\space {s}^{-1}{\mathrm{mM}}^{-1}$ is the relaxivity of Gadovist at 7T^28^. The effective ${T}_{1}$ was calculated by solving the spin-echo signal equation at each timepoint:

3 $$SI=k\rho \left(1-{e}^{\frac{-TR}{{T}_{1}}}\right){e}^{\frac{-TE}{{T}_{2}}}$$

where $k$ is a scaling constant and $\rho$ is the proton density. Assuming that ${T}_{2}\ggg TE$, the effective ${T}_{1}$ was calculated by dividing the signal intensity at time $t$ by the baseline signal intensity leading to $k$ and $\rho$ cancelling out.

Semi-quantitative analysis was performed on the resulting Gd time-concentration curves for each voxel^29^. The maximum enhancement (ME) was the maximum Gd concentration reached by the given voxel over the DCE-MRI time course. The time-to-peak Gd concentration (TTP) was defined as the time from initial contrast agent arrival in the voxel to the time at which maximum Gd concentration was reached. The wash-in rate (WIR) was calculated as the slope of the line connecting the point of initial contrast agent arrival in the voxel to the point of maximum Gd concentration. The area under the curve (AUC) was calculated over 15 min from initial contrast agent arrival in the voxel.

To progress beyond the empirical concentration curve analysis above, a variety of compartmental models have been proposed in the medical imaging community that offer more biophysical insight through the model fitting parameters. In this context, we performed a non-linear least-squares fitting of the Toft’s model^30^ to the Gd time-concentration curves for each voxel. This approach allowed for direct estimation of ${k}_{trans}$ which is the rate of Gd extravasation from the intravascular to the extravascular extracellular space (EES). The fractional volume of the EES (${v}_{e}$) was also calculated by fitting of the Toft’s model, expressed as

4 $$C_{t} \left( t \right) = k_{{trans}} \int_{0}^{t} {C_{p} \left( \tau \right){\text{exp}}\left[ { - \frac{{k_{{trans}} }}{{v_{e} }}(t - \tau )} \right]d\tau }$$

where ${C}_{t}$ is the Gd concentration at time $t$, and ${C}_{p}$ is the concentration of Gd in the blood plasma (arterial input function, AIF)^30^. A biexponential population-based AIF for mice proposed by Benjaminsen et al^21^ was used,

5 $${C}_{p}\left(t\right)={A}_{0}({a}_{1}{e}^{-{b}_{1}t}+{a}_{2}{e}^{-{b}_{2}t})$$

The constants in this equation were ${A}_{0}=0.75 \space\mathrm{mmol}/\mathrm{kg}$, ${a}_{1}=8.5\space\mathrm{ kg}/\mathrm{L}$, ${b}_{1}=4.8\space{\mathrm{min}}^{-1}$, ${a}_{2}=45\space\mathrm{kg}/\mathrm{L}$, and ${b}_{2}=0.06\space{\mathrm{ min}}^{-1}$ as measured previously by Benjaminsen *et al*^21^. The rate constant for Gd moving from the EES to the intravascular space (${k}_{ep}$) was determined using Eq. (6)^30^.

6 $${k}_{ep}=\frac{{k}_{trans}}{{v}_{e}}$$

### OCT-MRI correlations

MRI parameter maps were co-registered to the segmented svOCT vascular volumes using fiducial markers attached to the window chamber (Fig. 1A). Three different “sliding window” VOI sizes (lateral $\times$ lateral $\times$ axial—$0.5\times 0.5\times 1\space{\mathrm{mm}}^{3}, 1\space{\mathrm{mm}}^{3}, 1.5\times 1.5\times 1\space{\mathrm{mm}}^{3}$) were used to facilitate intermodality spatial correlations between the two co-registered datasets (Fig. 3B and C). The sliding window VOI was displaced in increments of $0.5\space\mathrm{mm}$, corresponding to the size of the DCE-MRI voxels. Intermodality correlations were only performed if at least 75% of the sliding window VOI was within the manually drawn tumor contour to reduce signal from ‘healthy’ tissue surrounding the tumor. For each position of the sliding window VOI, the average value of the DCE-MRI parameter map voxels was calculated (Fig. 3B and D) and directly compared with the VVF and $\overline{\mathrm{DNV} }$ calculated within the same VOI on the segmented svOCT dataset (Fig. 3C and E).

### Statistical analysis

All statistical analysis was performed in MATLAB R2020A (MathWorks, Inc., Natick, MA, USA). For *intramodality* comparison between normal and tumor tissue, a two-tailed Wilcoxon rank sum test was used. $P<0.05$ level was selected to indicate a statistically significant difference between the two groups.

To assess the strength of svOCT and DCE-MRI *intermodality* correlations, the DCE-MRI metrics for each position of the sliding window VOI were plotted against the corresponding svOCT measurements for the VOI. The Spearman’s correlation coefficient ($r$) was used to assess the strength of intermodality correlations. $P<0.05$ was considered statistically significant. Spearman correlation coefficients were reported and interpreted according to recent guidelines^31^.

## Results

### Healthy vs. tumor vasculature: quantification by svOCT and DCE-MRI

To validate our individual svOCT and MRI systems and compare our results with other studies, we performed an initial comparison of healthy and tumor tissue. Analysis of tumor-bearing and healthy mice was performed using a 1 ${\mathrm{mm}}^{3}$ sliding window VOI (see supplementary information Table S1 and Table S2 for results using the $0.5\times 0.5\times 1\space{\mathrm{mm}}^{3}$ and $1.5\times 1.5\times 1\space{\mathrm{mm}}^{3}$ VOI sizes respectively). Table 1 shows the average ± standard deviation values for the svOCT and DCE-MRI vascular metrics in tumor-bearing and healthy bare skin mice. n = 7 tumor bearing mice were included in the analysis. n = 2 healthy bare skin mice were used for calculation of the svOCT-derived metrics. n = 1 healthy bare skin mouse was used to derive the DCE-MRI metrics since MR imaging of the other healthy mouse was not successful due to the presence of exudate between the tissue and the glass of the window chamber, leading to partial volume artifacts. Average values and standard deviations were calculated based on the measurements from all positions of the sliding window VOI across all tumor-bearing or healthy mice.

**Table 1:** Healthy vs. tumor tissue quantification by svOCT and DCE-MRI.

		Tumor	Healthy	*P*-value
svOCT Vascular Metrics	Vascular Volume Fraction, VVF	$0.05\pm 0.04$	$0.23\pm 0.06$	$<0.0001$
	Mean Distance to Nearest Vessel, $\overline{\mathrm{DNV} }$ [$\mu \mathrm{m}$]	$200\pm 160$	$26\pm 5$	$<0.0001$
DCE-MRI Semi-Quantitative Metrics	Area Under the Curve, AUC [mM $\bullet$ min]	$3.91\pm 1.65$	$6.77\pm 1.63$	$<0.0001$
	Maximum Enhancement, ME [mM]	$0.38\pm 0.14$	$0.56\pm 0.14$	$<0.0001$
	Time to Peak, TTP [$\mathrm{min}$]	$14.83\pm 5.77$	$7.79\pm 1.29$	$<0.0001$
	Wash in Rate, WIR [${10}^{-2}$ mM/min]	$3.49\pm 1.82$	$7.85\pm 2.46$	$<0.0001$
DCE-MRI Fully-Quantitative Metrics	Volume Transfer Constant, ${k}_{trans}$ [${\mathrm{min}}^{-1}$]	$0.03\pm 0.02$	$0.11\pm 0.05$	$<0.0001$
	Fractional Volume of EES,${v}_{e}$	$0.26\pm 0.10$	$0.26\pm 0.07$	0.63
	Rate Constant from EES to Intravascular Space, ${k}_{ep}$ [${\mathrm{min}}^{-1}$]	$0.16\pm 0.16$	$0.42\pm 0.16$	$<0.0001$

Examining the svOCT metrics first, tumor bearing mice had a much lower vascular volume fraction (VVF) than healthy mice, indicating impaired vascular development consistent with the literature^11^. The VVF metric was measured in this analysis because it is useful, straightforward to calculate and widely cited^6,8,11,20–24^, thus also facilitating comparison to other studies. However, the 3D imaging capabilities of OCT enables derivation of additional metrics of potential biophysical utility, such as for example the mean distance to the nearest vessel ($\overline{\mathrm{DNV} }$). This may prove useful for monitoring the ability of the vascular network to deliver oxygen and nutrients to the surrounding cells^32–34^. $\overline{\mathrm{DNV} }$ has direct linkages to tumor cell hypoxia with regions >$100-150\space\mu\mathrm{m}$ (diffusion distance of oxygen) typically being (chronically) hypoxic^32,33^. The aggressive growth of tumor cells often leads to the formation of large avascular regions and thus a decrease in $\overline{\mathrm{DNV} }$ may also be associated with vascular normalization^34,35^. Not surprisingly, we found lower VVF and higher $\overline{\mathrm{DNV} }$ in tumor bearing mice than in normal controls (Table 1, top rows). These both indicate a decrease in the ability for the vasculature to deliver oxygen and nutrients to the surrounding cells, as well as the increased likelihood of hypoxic regions in the tumor-bearing mice.

**Figure 4:**
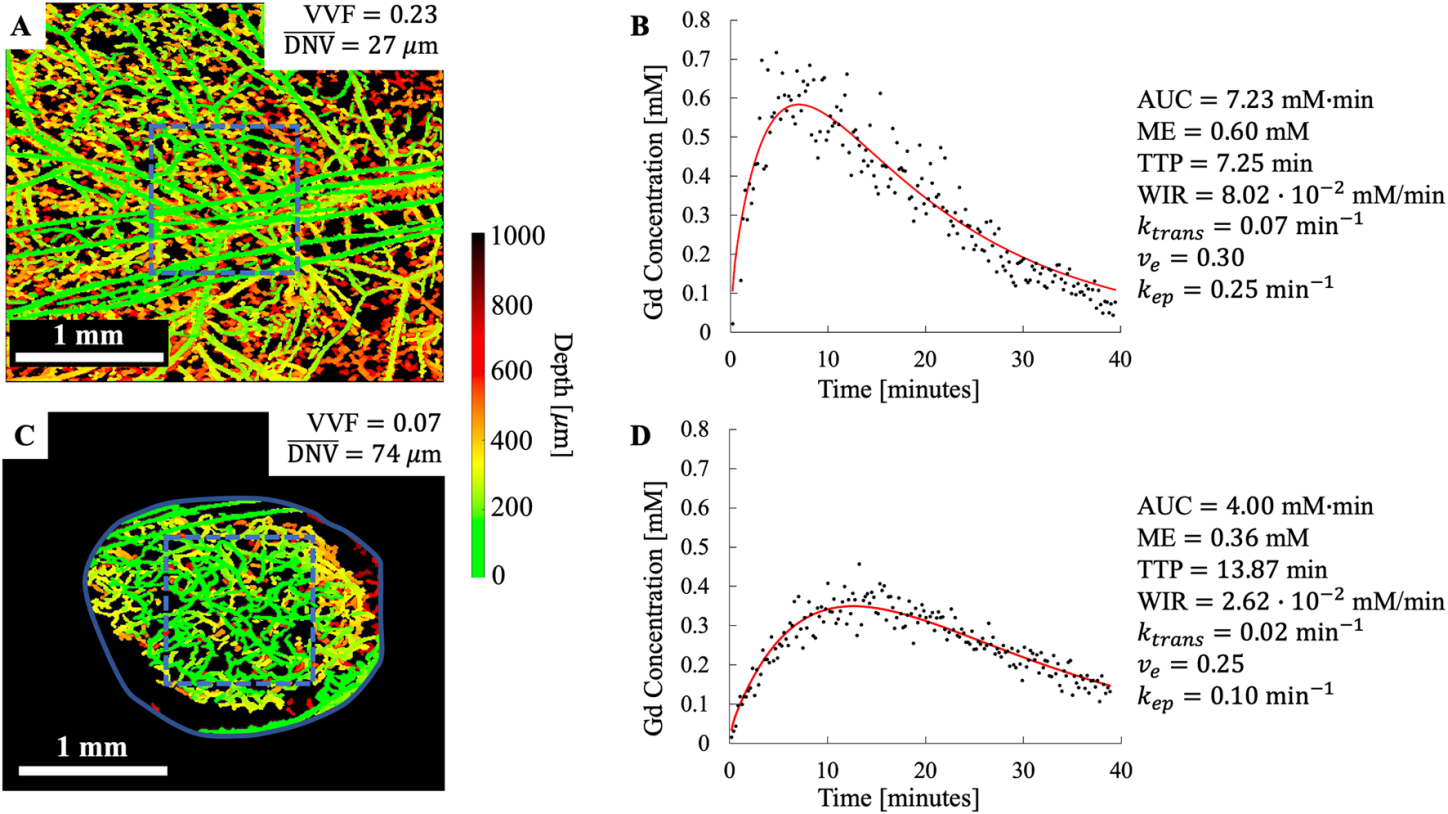
Healthy vs. tumor tissue quantification by svOCT and DCE-MRI. Significant differences in the microvasculature and corresponding DCE-MRI concentration–time curves were observed when comparing healthy and tumor tissue. (**A)** segmented depth-encoded svOCT microvascular map of healthy (bare skin) mouse and corresponding DCE-MRI Gd time concentration curve (**B**). (**C)** and (**D)** present analogous results for a tumor-bearing mouse. Gd time concentration curves and svOCT vascular metrics were calculated within a 1 ${\mathrm{mm}}^{3}$ volume of interest (blue dotted line) shown in (**A)** and (**C)**. The solid blue line in (**C)** shows the tumor contour and the red line in (**B)** and (**D)** are the Toft’s model fits to the data. (**A)** and (**C)** were generated using MATLAB R2020A software (MathWorks, Inc., Natick, MA, USA).

A statistically significant difference between healthy and tumor tissue was found for all semi-quantitative DCE-MRI metrics (Table 1, middle rows). The variation in semi-quantitative DCE-MRI metrics can be best explained by Fig. 4 which shows the svOCT vascular maps and corresponding DCE-MRI contrast enhancement curves for a healthy mouse (Fig. 4A and B) and tumor bearing mouse (Fig. 4C and D). The healthy mouse exhibited a high wash in rate, earlier TTP, larger ME, and larger AUC in comparison to tumor bearing mice. This rapid and early enhancement with a high ME is indicative of well perfused tissue with a high vascularity^36,37^. On the other hand, tumor bearing mice had a shallower wash in slope along with decreased ME and a longer TTP indicating decreased perfusion compared to healthy mice^36,37^.

The Toft’s model fit to the Gd time concentration curves are shown as the red line in Fig. 4B and D. ${k}_{trans}$ was higher in healthy mice than in tumor-bearing mice. ${k}_{trans}$ is dependent on tissue blood flow, vascular permeability, and capillary surface area^30^. Therefore, the larger ${k}_{trans}$ values in healthy mice may be attributed to the increased blood flow and vascular surface area in the healthy mice. This finding must be interpreted carefully however, since tumor vasculature is inherently ‘leaky’ which could contribute to higher ${k}_{trans}$ values in the tumor tissue in comparison to healthy tissue depending on the tumor type. ${v}_{e}$ was not different between healthy and tumor-bearing mice indicating that the cell density was similar in healthy and tumor tissue. ${k}_{ep}$ showed the same trend as ${k}_{trans}$; ${k}_{ep}$ was larger in healthy tissue compared to tumor tissue as expected since it is directly related to ${k}_{trans}$ by Eq. (6).

Overall, comparison of healthy and tumor tissue showed significant differences in microvascular structure (as shown by svOCT) as well as differences observed on DCE-MRI contrast enhancement curve shape and fully quantitative tissue parameters. The delayed contrast enhancement with shallower wash in rate and decreased maximum enhancement is consistent with impaired vascular development in tumors compared to healthy controls. Fully quantitative metrics displayed significant differences in ${k}_{trans}$ values between healthy and tumor tissue, however the influence of vascular permeability (tumor leakiness) may have confounding effects.

### Micro svOCT to macro DCE-MRI vascular comparisons

To determine which DCE-MRI macrometrics may offer the most insight into the underlying tissue microvasculature as reported by svOCT, a $1$ ${\mathrm{mm}}^{3}$ sliding window VOI (see Fig. 3) was used to quantify intermodality correlations (see supplementary information Table S3 and Table S4 for results using the $0.5\times 0.5\times 1\space{\mathrm{mm}}^{3}$ and $1.5\times 1.5\times 1\space{\mathrm{mm}}^{3}$ VOI sizes respectively). The results are summarized in Table 2, for both the healthy control (n = 1) and tumor-bearing (n = 7) mice.

**Table 2:** Spearman correlation coefficients for svOCT and DCE-MRI comparisons.

		DCE-MRI: Semi-Quantitative Metrics				DCE-MRI: Fully-Quantitative (Toft’s Model) Metrics		
		AUC	TTP	WIR	ME	${k}_{trans}$	${v}_{e}$	${k}_{ep}$
svOCT: Vascular Volume Fraction (VVF)	$r$	0.50	− 0.81	0.59	0.28	0.64	− 0.21	0.71
	*P*-value	$<0.0001$	$<0.0001$	$<0.0001$	$0.0010$	$<0.0001$	$0.0148$	$<0.0001$
svOCT: Mean Distance to Nearest Vessel ($\overline{\mathrm{DNV} }$)	$r$	− 0.48	0.83	− 0.57	− 0.26	− 0.61	0.26	− 0.70
	*P-*value	$<0.0001$	$<0.0001$	$<0.0001$	$0.0024$	$<0.0001$	$0.0023$	$<0.0001$

Firstly, the magnitude of Spearman correlation coefficients between DCE-MRI metrics and svOCT metrics (VVF and $\overline{\mathrm{DNV} }$) were essentially identical in magnitude and of opposite sign. For example, the Spearman correlation coefficient between ${k}_{ep}$ and VVF was $0.71$, and for ${k}_{ep}$ and $\overline{\mathrm{DNV} }$ it was $-0.70$. This implies that VVF and $\overline{\mathrm{DNV} }$ may in fact not be independent metrics of vascular microarchitecture. To check this, we plotted $\overline{\mathrm{DNV} }$ vs. VVF on a log–log plot (supplementary information Fig. S1). Linear regression analysis showed that VVF and $\overline{\mathrm{DNV} }$ were highly (anti)correlated (${R}^{2}=0.88$). The slope of the best fit line on the log–log plot was determined to be $-0.96$ indicating that:

7 $$\mathrm{VVF}\propto {\overline{\mathrm{DNV}} }^{-0.96}$$

This makes sense, in that increasing vascular content (large VVF) implies decreasing intervascular spaces (small $\overline{\mathrm{DNV} }$).

**Figure 5:**
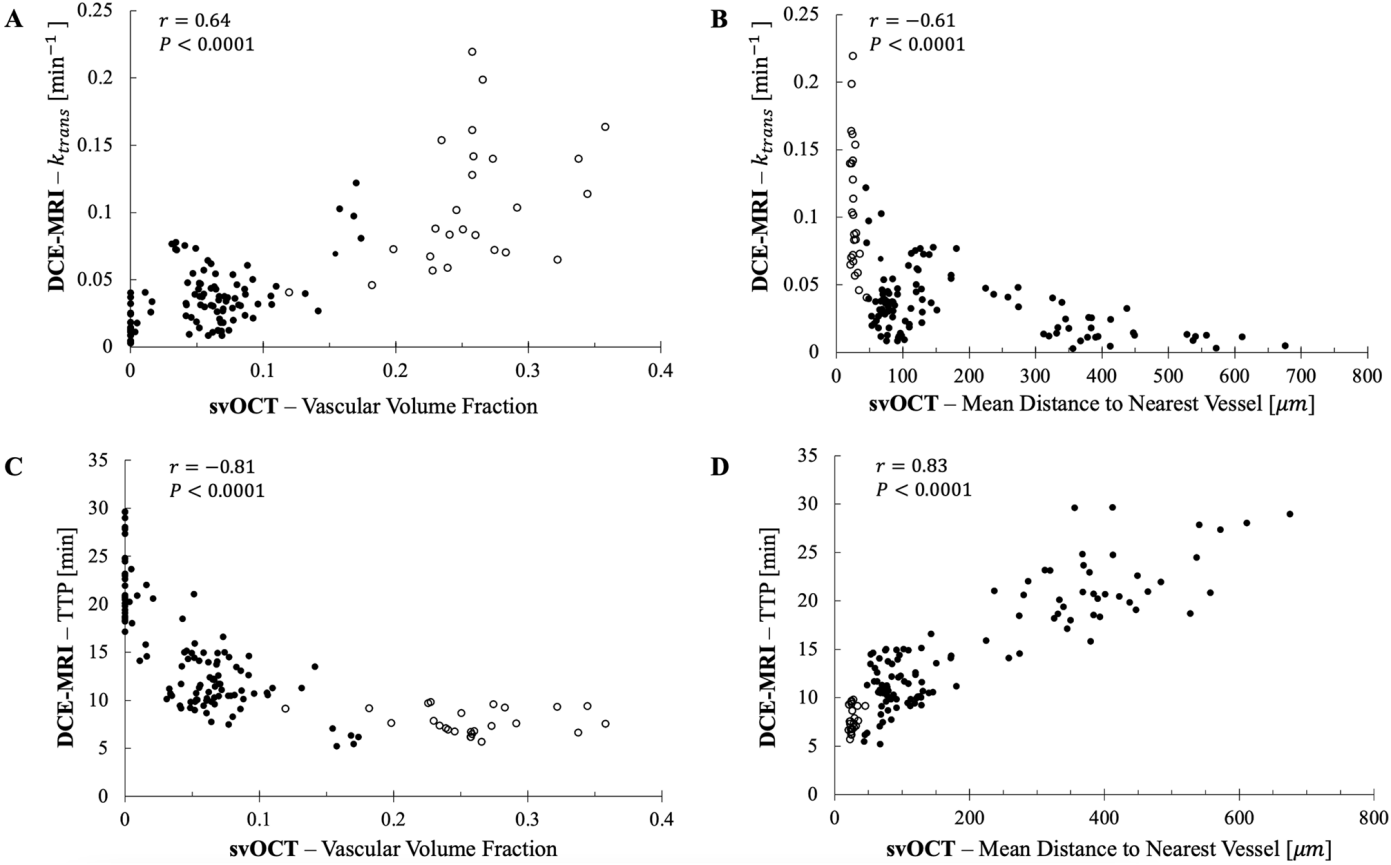
Notable ‘macro-to-micro’ pairs. (**A)** and (**B)** are correlation plots for MR’s ${k}_{trans}$ metric with svOCT-derived microvascular biomarkers VVF and $\overline{\mathrm{DNV} }$ respectively. (**C)** and (**D)** show analogous results for MR’s TTP metric. Each point represents the values obtained from the co-registered DCE-MRI and svOCT datasets for a single position of the $1\space{\mathrm{mm}}^{3}$ sliding window VOI. $r$ values = Spearman’s correlation coefficient. Open and solid symbols = healthy (n = 1) and tumor-bearing (n = 7) mice, respectively.

Examining microvascular svOCT correlations with the DCE-MRI *fully*-quantitative parameters first, moderate correlations were noted for ${k}_{trans}$ with VVF and $\overline{\mathrm{DNV} }$ ($r=0.64$ and $-0.61$ respectively, $P<0.0001$ for both) (Fig. 5A and B). Correlations between ${k}_{trans}$ and VVF are consistent with other window chamber and histology-based studies that compare vascular density to ${k}_{trans}$^13,14,21,24^. This positive correlation is expected since ${k}_{trans}$ is dependent on tissue blood flow, vascular permeability, and capillary surface area^30^. Therefore, the positive correlation with VVF (and negative correlation with $\overline{\mathrm{DNV} }$) can likely be attributed to the increase in tissue blood flow and/or vascular surface area as VVF increases (and $\overline{\mathrm{DNV} }$ decreases).

A negligible correlation was found for ${v}_{e}$ with VVF and $\overline{\mathrm{DNV} }$ ($r=-0.21, P=0.0148$ and $r=0.26 , P=0.0023$ respectively) which is consistent with histological studies that found no relationship with mean vascular density and ${v}_{e}$^24^. ${k}_{ep}$ was highly correlated with VVF and $\overline{\mathrm{DNV} }$ ($r=0.71$ and $-0.70$ respectively, $P<0.0001$ for both) however this high correlation may be attributed to the ${k}_{trans}$ parameter since ${k}_{ep}$ is directly related to ${k}_{trans}$ by Eq. (6).

Moving on to microvascular svOCT correlations with the DCE-MRI *semi*-quantitative parameters, a high correlation was found for TTP with VVF and $\overline{\mathrm{DNV} }$ ($r=-0.81$ and $0.83$ respectively, $P<0.0001$ for both) (Fig. 5C and D). This negative relationship between TTP and VVF has also been identified in some previous histology studies^22,23^. A moderate correlation was found for WIR with VVF and $\overline{\mathrm{DNV} }$ ($r=0.59$ and $-0.57$ respectively, $P<0.0001$ for both). However, the correlation of WIR with svOCT microvascular metrics must be interpreted carefully. WIR is directly related to TTP and ME by:

8 $$\mathrm{WIR}=\frac{\mathrm{ME}}{\mathrm{TTP}}$$

Note that the numerator in Eq. (8) is ME since the baseline Gd concentration is ~ 0. A negligible correlation between ME with VVF and $\overline{\mathrm{DNV} }$ ($r=0.28, P=0.0010;$ $r=-0.26, P=0.0024$ respectively) was noted. Therefore, the moderate correlation with WIR and svOCT microvascular parameters may likely be attributed to the TTP metric.

Interestingly, low to moderate correlations were noted for the semi-quantitative MRI metric, AUC ($r=0.50$ and $-0.48$ respectively, $P<0.0001$ for both). Many prior studies have identified a high correlation between AUC and ${k}_{trans}$^38,39^, which may explain these findings. Changing the time over which AUC is integrated over may also lead to improved correlations with svOCT microvascular metrics.

## Discussion

The 3D high resolution imaging capabilities of svOCT allowed for accurate vascular microarchitecture metric extraction, and the subsequent co-registration with DCE-MRI time series datasets allowed for spatial correlation analysis between the two modalities in same live animals. The correlations between TTP, ${k}_{trans}$, and AUC, if verified in further studies, suggest that DCE-MRI can be used clinically to accurately quantify tissue microvasculature. That is, it may become possible to associate MR’s macrovascular dynamic biomarkers with microvascular architectural features beyond their current spatial resolution limit. Interestingly, MR’s semi-quantitative metrics were comparable (or better) to fully quantitative metrics in quantifying the microvasculature (Table 2). This finding is potentially of great interest since semi-quantitative metrics may be more robust, and reproducible than fully quantitative metrics^40,41^.

The highest correlation identified in our study was TTP with svOCT microvascular metrics VVF and $\overline{\mathrm{DNV} }$ ($r=-0.81$ and $0.83$ respectively, $P<0.0001$ for both) (Fig. 5C and D). To better understand this relationship, we substituted Eq. (5) into Eq. (4). Setting the time derivative of the resulting expression to zero to find the TTP, in the limit where ${b}_{1}\cdot {\text{TTP}}\gg 1$ (expected to generally be the case since the initial decay rate ${b}_{1}$ is typically on the order of ten minutes^−1^, including in human patients^42,43^), TTP is approximated by:

9 $$\text{TTP} \approx \frac{{\text{ln}}\left({k}_{ep}/{b}_{2}\right)}{{k}_{ep}-{b}_{2}}$$

In both the flow- and permeability-limited regimes of tracer transport, ${k}_{trans}$ and hence, ${k}_{ep}$, is proportional to VVF^30^. In combination with Eq. (7), TTP may thus be a sensitive surrogate for svOCT parameters. Although TTP also depends on the long-time clearance rate ${b}_{2}$ of tracer from the blood (Eq. (9)), this quantity should be much easier to determine in a patient-specific manner than the full AIF, since it can be extracted from the portion of the AIF where Gadolinium concentration is low and hence, less susceptible to AIF quantification errors stemming from bolus dispersion effects^44^. The TTP is also not sensitive to our choice of bi-exponential AIF form since in the expected limit where the fast clearance dynamics (e.g., ${b}_{1}$) are much faster than 1/TTP, TTP is only sensitive to the long-time clearance dynamics, well-described by a single exponential term.

These findings plus the high correlation between VVF and *mean* DNV ($\overline{\mathrm{DNV} }$) (Fig. S1 and Eq. (7)) suggest that DCE-MRI using small molecular-weight tracers, such as Gadovist, may not be sensitive to properties of the DNV histogram (intra-voxel distribution of DNV values) that may be most relevant for hypoxia and vascular transport efficiency^32–34^. A hallmark of irregular tumor vasculature is the presence of aperfused regions, giving rise to a long tail in the DNV histogram at high DNV values^34,45^. This is important for agents such as oxygen and large molecular-weight chemotherapy agents, for which transport is diffusion-limited, characterized by equilibration times (e.g., TTP) that scale as the *square* of the DNV: DNV^2^/*D*, where *D* is the agent diffusivity^34^. In moving from micron (OCT) to mm (MRI voxel) scales, this square dependence enhances the contribution of the aperfused regions (large DNV) in the voxel average. Conversely, the *mean* DNV ($\overline{\mathrm{DNV} }$) value is less sensitive to the presence of these regions. This is reflected in Eq. (7): for an array of capillary “rods” with zero tortuosity, one can show that ${\text{VVF}}\propto {\overline{\mathrm{DNV}} }^{-1/2}$. The deviation of the measured exponent from 1/2 thus reflects the tortuosity of the vascular array but is otherwise insensitive to the DNV histogram. We speculate that higher molecular-weight and liposomal MRI contrast agents^46^, with slower diffusivity, will exhibit transport properties more sensitive to the DNV histogram and hence may be better suited to bridging the macro to micro resolution gap between the full suite of svOCT microvascular pathology metrics and clinical imaging modalities.

Previous studies have validated DCE-MRI parameter maps by directly comparing MRI measurements of tissue vascularity with histological measurements of the vascular density, a 2D *ex-vivo* analogue of our *in-vivo* 3D VVF metric^20–24^. These studies are limited by their 2-dimensional analysis of vascular density using histological preparations which are also subject to uncertainty related to the number and location of histological sections taken from the tumor. Others report confocal microscopy images of the tumor vasculature to compare with DCE-MRI parameter maps^13,14^ but these are also limited by their 2-dimensional analysis of the vasculature. Our advanced svOCT imaging platform allows for high resolution 3D images of microvasculature *in-vivo*. This provides a more accurate assessment of the VVF and $\overline{\mathrm{DNV} }$. Future work will derive additional potentially useful microvascular metrics from our 3D svOCT datasets such as tortuosity and fractal dimension for direct correlation with MRI^3,47^. Our ability to co-register these 3D svOCT vascular images with DCE-MRI datasets *in-vivo* in the same animals adds an “extra level of realism” to these studies. The spatial correlations that we have performed are important for demonstrating the ability of DCE-MRI to differentiate between regions in the same tumor that have different vascular microstructures. This may prove useful in several clinical scenarios including radiotherapy applications where conformal doses can be created to selectively boost or avoid specific regions of the tumor (i.e. dose painting)^48^. For example, conformal radiotherapy plans may be created using these spatial DCE-MRI parameter maps to selectively avoid poorly vascularized tumor regions (i.e. regions with a large $\overline{\mathrm{DNV} }$ or small VVF), to allow for revascularization and reoxygenation of those tumor cells while still treating regions of the tumor that are well vascularized.

Multiple sliding window VOI sizes were investigated to perform OCT-MRI correlations. The smallest VOI corresponded to the size of MRI voxels in the window chamber plane ($0.5\times 0.5\times 1\space{\mathrm{mm}}^{3}$). A $1\space{\mathrm{mm}}^{3}$ VOI (results presented) and a $1.5\times 1.5\times 1 \space{\mathrm{mm}}^{3}$ VOI were also used (see supplementary information Table S3 and Table S4 respectively). There was a general improvement in the intermodality correlations with increasing VOI size. This can likely be attributed to slight 3D mis-registration between the svOCT and DCE-MRI datasets which may cause larger artefacts as VOI size decreases.

An important variable and a potential source of error in this study was the definition of the tumor margin in the svOCT datasets. The tumor contour has some variability as it was drawn manually based on the structural OCT scans and the co-registered DsRed fluorescence datasets (which highlights the viable tumor cell compartment). This made it challenging to define the tumor boundary below the surface of the tissue, especially in situations where there was exudate buildup in the window chamber. To reduce this uncertainty, future work will implement a 3D tumor segmentation algorithm we are currently refining that is more objective (based solely on texture analysis of the structural OCT scans)^49^.

In conclusion, we have performed in vivo 3D microvascular imaging using high resolution svOCT and comparably lower resolution DCE-MRI via dual-modality-compatible window chamber mouse xenograft tumor model. Our goal was to use the detailed volumetric visualization afforded by svOCT to increase the information content derivable from clinically measurable DCE-MRI metrics, towards understanding / optimizing / guiding feedback-driven adaptive vascular targeting therapies. We thus performed co-registered spatial correlation analysis between svOCT microvascular descriptors (VVF and $\overline{\mathrm{DNV} }$) and DCE-MRI’s semi- and fully- quantitative macrovascular metrics. Various macro-to-micro vascular linkages were identified between the two modalities, and their respective degrees of correlation were quantified. For example, the noted high correlation between svOCT’s $\overline{\mathrm{DNV} }$ and VVF with MR’s TTP metric and moderate to low correlation with ${k}_{trans}$ and AUC metrics makes sense biologically and adds previously unattainable important information content to the MR vascular quantification toolbox. Overall, the presented methodology for bridging the macro-to-micro resolution gap in angiography may prove useful for tissue functional assessment and therapeutic response monitoring towards treatment optimization and personalization.

## Supplementary Information

Supplementary Information.

## Data Availability

The datasets generated and analysed during this study are available from the corresponding author upon reasonable request.
