# Supplementary material for: Bridging the macro to micro resolution gap with angiographic optical coherence tomography and dynamic contrast enhanced MRI
Source: Sci Rep. 2022 Feb 24;12:3159. doi: 10.1038/s41598-022-07000-1 (PMC8873467; doi:10.1038/s41598-022-07000-1)
Supplement: Supplementary file 1 — Supplementary Information. [file 41598_2022_7000_MOESM1_ESM.pdf]

Supplementary Information for:

Bridging the Macro to Micro Resolution Gap with Angiographic Optical Coherence Tomography and Dynamic Contrast Enhanced MRI

W. Jeffrey Zabel[1,*,†] , Nader Allam[1,†], Warren D. Foltz[2,3], Costel Flueraru[4], Edward Taylor[2,3], I. Alex Vitkin[1,2,3].

[1]Department of Medical Biophysics, University of Toronto, Toronto, Canada.
[2]Radiation Medicine Program, Princess Margaret Cancer Centre, Toronto, Canada.
[3]Department of Radiation Oncology, University of Toronto, Toronto, Ontario.
[4]National Research Council Canada, Information Communication Technology, Ottawa, Canada.
[*]Corresponding Author: jeff.zabel@mail.utoronto.ca.
[†]These authors contributed equally to this work

		Tumor	Healthy	P-value
svOCT Vascular Metrics	Vascular Volume Fraction, VVF (%)	0.05 ± 0.05	0.24 ± 0.08	< 0.0001
	Mean Distance to Nearest Vessel, $\overline{\text{DNV}}$ [μm]	186 ± 161	26 ± 8	< 0.0001
DCE-MRI Semi-Quantitative Metrics	Area Under the Curve, AUC [mM·min]	4.15 ± 2.18	6.58 ± 2.17	< 0.0001
	Maximum Enhancement, ME [mM]	0.40 ± 0.19	0.55 ± 0.18	< 0.0001
	Time to Peak, TTP [min]	14.43 ± 6.11	8.06 ± 1.62	< 0.0001
	Wash in Rate, WIR [10^{-2} mM/min]	3.76 ± 2.35	7.34 ± 3.05	< 0.0001
DCE-MRI Fully-Quantitative Metrics	Volume Transfer Constant, k_{trans} [min^{-1}]	0.04 ± 0.03	0.09 ± 0.06	< 0.0001
	Fractional Volume of EES, v_e	0.26 ± 0.12	0.26 ± 0.10	0.96
	Rate Constant from EES to Intravascular Space, k_{ep} [min^{-1}]	0.18 ± 0.19	0.38 ± 0.18	< 0.0001

Supplementary Table S1. $0.5 \times 0.5 \times 1$ mm^3 sliding VOI comparison of healthy and tumor tissue.

		Tumor	Healthy	P-value
svOCT Vascular Metrics	Vascular Volume Fraction, VVF (%)	0.05 ± 0.04	0.26 ± 0.08	< 0.0001
	Mean Distance to Nearest Vessel, $\overline{\text{DNV}}$ [μm]	205 ± 159	25 ± 4	< 0.0001
DCE-MRI Semi-Quantitative Metrics	Area Under the Curve, AUC [mM·min]	3.79 ± 1.45	6.84 ± 1.02	< 0.0001
	Maximum Enhancement, ME [mM]	0.37 ± 0.12	0.57 ± 0.09	< 0.0001
	Time to Peak, TTP [min]	14.90 ± 5.46	7.60 ± 0.98	< 0.0001
	Wash in Rate, WIR [10^{-2} mM/min]	3.34 ± 1.64	8.14 ± 1.66	< 0.0001
DCE-MRI Fully-Quantitative Metrics	Volume Transfer Constant, k_{trans} [min^{-1}]	0.03 ± 0.02	0.11 ± 0.03	< 0.0001
	Fractional Volume of EES, v_e	0.24 ± 0.09	0.26 ± 0.05	0.17
	Rate Constant from EES to Intravascular Space, k_{ep} [min^{-1}]	0.16 ± 0.12	0.45 ± 0.12	< 0.0001

Supplementary Table S2. $1.5 \times 1.5 \times 1$ mm^3 sliding VOI comparison of healthy and tumor tissue.

		DCE-MRI: Semi-Quantitative Metrics				DCE-MRI: Fully-Quantitative (Toft's Model) Metrics		
		AUC	TTP	WIR	ME	k_{trans}	v_e	k_{ep}
svOCT: Vascular Volume Fraction (VVF)	r	0.44	-0.74	0.53	0.26	0.61	-0.14	0.65
	P-value	< 0.0001	< 0.0001	< 0.0001	0.0004	< 0.0001	0.0556	< 0.0001
svOCT: Mean Distance to Nearest Vessel ($\overline{\text{DNV}}$)	r	-0.42	0.74	-0.50	-0.21	-0.56	0.19	-0.62
	P-value	< 0.0001	< 0.0001	< 0.0001	0.0032	< 0.0001	0.0124	< 0.0001

Supplementary Table S3. Spearman correlation coefficients for svOCT and DCE-MRI comparisons
NOTE: A $0.5 \times 0.5 \times 1$ mm^3 sliding window VOI was used for svOCT and DCE-MRI correlation analysis.

		DCE-MRI: Semi-Quantitative Metrics				DCE-MRI: Fully-Quantitative (Toft's Model) Metrics		
		AUC	TTP	WIR	ME	k_{trans}	v_e	k_{ep}
svOCT: Vascular Volume Fraction (VVF)	r	0.57	-0.84	0.64	0.33	0.67	-0.13	0.69
	P-value	< 0.0001	< 0.0001	< 0.0001	0.0017	< 0.0001	0.2682	< 0.0001
svOCT: Mean Distance to Nearest Vessel ($\overline{\text{DNV}}$)	r	-0.56	0.85	-0.62	-0.33	-0.64	0.18	-0.68
	P-value	< 0.0001	< 0.0001	< 0.0001	0.0022	< 0.0001	0.1107	< 0.0001

Supplementary Table S4. Spearman correlation coefficients for svOCT and DCE-MRI comparisons
NOTE: A $1.5 \times 1.5 \times 1$ mm^3 sliding window VOI was used for svOCT and DCE-MRI correlation analysis.

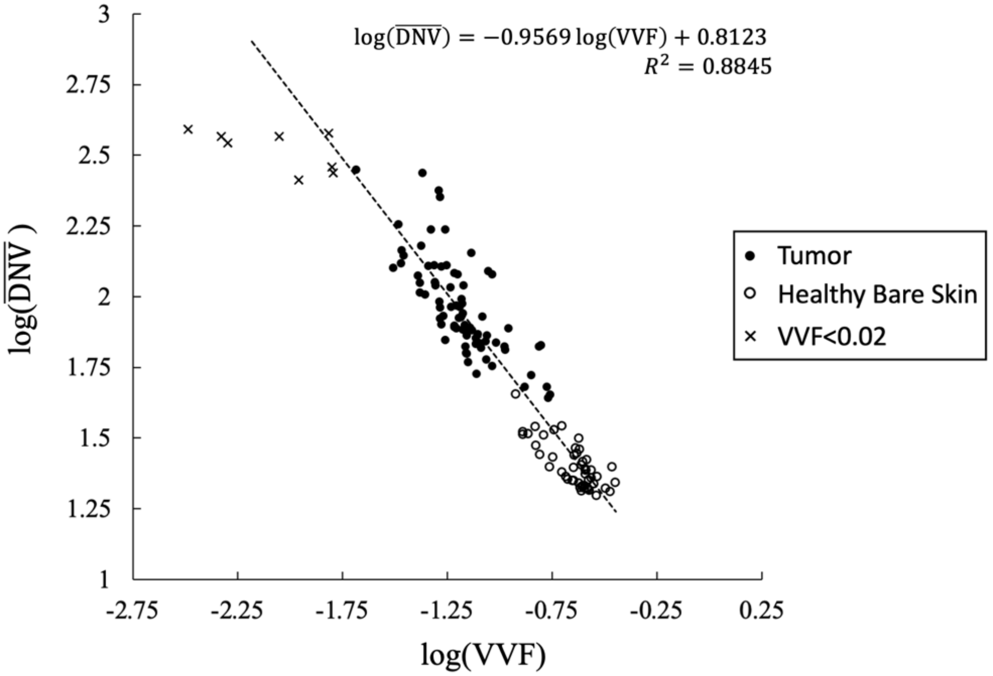

Supplementary Figure S1. Relationship between svOCT's microvascular biomarkers, $\overline{\text{DNV}}$ and VVF. The log-log plot of the vascular volume fraction (VVF) and mean distance to nearest vessel ($\overline{\text{DNV}}$) in healthy and tumor bearing mice was fitted with a straight line using the linear least squares approach ($R^2 = 0.88$). Each point on this plot represents the values obtained from one position of a $1\ \text{mm}^3$ sliding VOI across all mice. The slope of the line of best fit line was -0.96, indicating that VVF $\propto \overline{\text{DNV}}^{-0.96}$. Points with a VVF < 0.02 (cross points on plot) were excluded from the fit since they were heavily influenced by VOI size and position in proximity to a vascularized 'rim' of healthy tissue surrounding some largely avascular tumors. Open symbols = healthy mice (n=2); solid symbols = tumor-bearing mice (n=7).
